# Development and Exploratory Validation of the Clinical Research Nursing Competencies-Self-Efficacy Scale

**DOI:** 10.3390/healthcare14040551

**Published:** 2026-02-23

**Authors:** Mattia Bozzetti, Laura Apadula, Arianna Magon, Gianluca Conte, Daniele Napolitano, Giulia Villa, Monica Guberti, Rosario Caruso

**Affiliations:** 1 Department of Biomedicine and Prevention, University of Rome Tor Vergata, 00133 Rome, Italy; 2 Pancreato-Biliary Endoscopy and Endosonography Division, Clinical Research Centre, Pancreas Translational and Clinical Research Center, IRCCS San Raffaele Scientific Institute, 20132 Milan, Italy; apadula.laura@hsr.it; 3 Health Professions Research and Development Unit, IRCCS Policlinico San Donato, 20097 San Donato Milanese, Italy; arianna.magon@grupposandonato.it; 4 Clinical Research Service, IRCCS Policlinico San Donato, 20097 San Donato Milanese, Italy; gianluca.conte@grupposandonato.it; 5 SITRA—Scientific Direction, Fondazione Policlinico Gemelli, 00168 Rome, Italy; daniele.napolitano@policlinicogemelli.it; 6 Center for Nursing Research and Innovation, Faculty of Medicine and Surgery, Vita-Salute San Raffaele University, 20132 Milan, Italy; villa.giulia@hsr.it; 7 Allied Health Professions Directorate, Orthopaedic Institute Rizzoli IRCCS, 40136 Bologna, Italy; monica.guberti@ior.it; 8 Department of Biomedical Sciences for Health, University of Milan, 20133 Milan, Italy; 9 Health Professions Research and Evidence Transfer Unit, IRCCS MultiMedica, 20099 Sesto San Giovanni, Italy

**Keywords:** clinical research, nursing competencies, psychometrics, self-efficacy, clinical trials

## Abstract

**Highlights:**

**What are the main findings?**

- A new, theory-grounded self-efficacy scale for Clinical Research Nursing (Se-CRN) was developed and validated, supporting a five-domain structure aligned with core CRN practice areas.
- Competency-related self-efficacy was most strongly associated with research-setting experience (and, to a lesser extent, advanced education), with comparatively lower confidence in study management and scientific contribution domains.

**What are the implications of the main findings?**

- The Se-CRN can support targeted training needs assessment, curriculum design, and workforce planning by identifying domain-specific strengths and gaps in CRN perceived capability.
- The scale enables evaluation of professional development pathways and could be used to monitor growth in CRN competence-related self-efficacy across settings and roles.

**Abstract:**

**Background/Objectives**: The objective of this study was to develop and validate the Clinical Research Nursing Competencies–Self-Efficacy (Se-CRN) scale, a theory-grounded instrument to assess perceived capability in clinical research nursing practice. **Methods**: A two-phase validation study was conducted using an exploratory sequential mixed-method design between July 2022 and September 2025. The initial item pool was derived from an established competency taxonomy and refined through expert review for face and content validity. The final version of the Se-CRN was administered online to Clinical Research Nurses working with patients enrolled in clinical trials in Italy. Structural validity was examined using exploratory factor analysis with parallel analysis, and reliability was assessed through internal consistency and hierarchical indices. Group differences in self-efficacy were examined across clinical settings, educational levels, and research experience. **Results**: A total of 183 nurses participated. The data supported a five-factor solution reflecting core dimensions of clinical research nursing (Clinical Practice, Study Management, Human Subject Protection, Contributing to the Science, and Care Coordination and Continuity). Reliability was excellent at the scale level and strong across domains. No differences in perceived capability were observed between oncological and non-oncological settings. Higher self-efficacy was consistently associated with greater experience in the research setting and, to a lesser extent, with advanced education. **Conclusions**: The Se-CRN is the first validated self-efficacy instrument that captures the full scope of clinical research nursing practice. It provides a practical measure to support training needs assessment, curriculum development, and workforce planning. Further research should confirm the factor structure and examine responsiveness to professional development across diverse settings.

## 1. Introduction

Clinical research nursing is defined as the specialized practice of nursing that balances the protection of research participants with the fidelity to study protocols. Clinical research nursing represents a unique intersection between biomedical science and patient-centered care [1]. In accordance with this dual mandate, CRN competence must be both adequate and dynamically updated to safeguard the quality and safety of both patients and research [2,3]. At the same time, the next decade will see rising complexity in healthcare delivery, driven by technological innovation and the diversification of care settings, requiring evolving competency sets [4]. These pressures are especially acute for Clinical Research Nurses (CRNs), who must navigate trials that are becoming more complex in design and conduct—spanning multisite coordination, data-intensive platforms, and rigorous regulatory requirements—while maintaining high-quality patient care and protocol fidelity [5]. Against this backdrop, well-prepared and motivated nurses become indispensable for delivering safe and effective care across all levels of the health system [6]. Critically, where nurse shortages persist, organizations triage scarce staff toward mandated routine and acute care [7]; research functions are among the first to be curtailed [8]. These dynamics are exacerbated by short-term, project-based contracts and limited formal recognition of the CRN role, which make research capacity particularly vulnerable during staffing crises [9].

Within this evolving landscape, CRNs must develop the capacity to recognize their knowledge gaps and professional development needs and to cultivate self-efficacy [10,11]. Nursing self-efficacy could be considered a reliable proxy for nurses’ work performance [12,13]. Self-efficacy is a core construct of social cognitive theory that denotes beliefs about one’s capability to perform task-specific activities and to manage challenging situations [14,15]. It is a modifiable predictor of performance that can be enhanced through targeted educational and motivational strategies, and it mediates the translation of knowledge into action. Self-efficacy is foundational to nursing practice and education because it can influence performance by influencing behaviors [12]. In this sense, domain-specific self-efficacy offers a theory-grounded, measurable indicator of competency development and readiness for increasingly complex trial work.

Beyond competence development, self-efficacy is embedded in broader psychosocial processes that shape nurses’ well-being, role functioning, and workforce sustainability. Recent evidence highlights how emotional functioning and psychological resources (such as emotional intelligence and perceived efficacy) relate to burnout and well-being in nursing populations and can be strengthened through structured learning experiences [16,17]. Complementary work from European settings also indicates that moral stressors and moral injury can erode nurses’ well-being and contribute to disengagement and turnover-related outcomes [18].

Yet, notwithstanding the availability of national and international competency frameworks [19,20,21], the evidence base underpinning their implementation remains mainly descriptive. A recent scoping review found that, although these frameworks comprehensively catalogue role domains and skills, institutional uptake is inconsistent, and psychometric or outcome-based evaluations are sparse or non-existent [9]. Most existing studies have focused on mapping CRN activities in terms of frequency and perceived importance, rather than investigating competence [22,23,24,25]. Moreover, the literature seldom links CRN competencies to patient-level outcomes and continues to document role ambiguity and organizational barriers that impede systematic evaluation [9]. This evidence gap underscores the need for empirically tested measures that operationalize competency-related constructs and enable hypothesis-driven studies on training, role development, and impact.

Thus far, no available tool has been designed or psychometrically tested to capture self-efficacy as a theory-grounded proxy of CRN competence across the full scope of research activities [9]. Existing measures typically assess generic professional confidence or focus on isolated task domains, providing limited value for evaluating readiness to manage increasingly complex trials or for guiding targeted professional development. Without robust self-efficacy metrics, educational interventions cannot be rigorously assessed, competency gaps cannot be systematically monitored, and the contribution of CRNs to research quality and participant safety remains under-operationalized [26]. For these reasons, this study reports the development and psychometric evaluation of the Clinical Research Nursing Competencies–Self-Efficacy (Se-CRN) scale, theoretically grounded in the Bevans–Castro taxonomy [20].

In this study, we aimed to develop and conduct an initial psychometric evaluation of the Se-CRN scale, a theory-grounded and practice-relevant instrument designed to assess CRNs’ self-efficacy across competency domains relevant to increasingly complex clinical trials. By operationalizing an established CRN competency taxonomy into measurable self-efficacy domains, we also provide empirical support for the coherence and measurability of these domains in an Italian CRNs sample.

## 2. Materials and Methods

### 2.1. Design

A validation study was conducted from July 2022 to September 2025, comprising two sequential phases within an exploratory sequential mixed-method design [27]. The first phase involves scale derivation, including construct definition and item generation, expert consultation and item revision, quantitative content validity analysis, and pilot testing for comprehensibility. The second phase consists of a cross-sectional study for psychometric evaluation. This study is reported in accordance with the Strengthening the Reporting of Observational Studies in Epidemiology (STROBE) guidelines for cross-sectional studies [28].

#### 2.1.1. Theoretical Framework and Item Pooling

According to Castro et al. [20], the conceptualization of the role draws on the five theoretical dimensions originally proposed by Hastings [21]: (1) clinical practice, (2) study management, (3) care coordination and continuity, (4) human subject protection, and (5) contributing to the science.

To operationalize these domains, 51 competencies for each dimension were developed and subsequently validated using the Delphi technique. The competencies were distributed as follows: clinical practice (4 activities), study management (24 activities), care coordination and continuity (9 activities), human subject protection (6 activities), and contributing to the science (8 activities). Based on the validated taxonomy and its corresponding competencies, the first version of the Se-CRN instrument was developed, comprising 51 items that reflect the various functions performed by CRNs in their professional roles. A back-translation procedure [29] was conducted for all items, and two were modified to ensure conceptual and contextual equivalence with the Italian National Health Service. During the item pooling phase, collegial discussions among subject-matter experts were held to critically examine and synthesize findings from the literature review [9], aiming to reach consensus on the most relevant competencies defining the CRN role. Based on this consensus, the conceptual dimensions underlying the Se-CRN questionnaire were confirmed, and an initial item pool was identified for inclusion in the instrument’s development.

#### 2.1.2. Face and Content Validity

Following the identification of the item pool, a panel of domain experts—comprising both methodological experts (nurse researchers and university faculty) and content experts (experienced CRNs)—was invited to assess the pertinence and relevance of each item for inclusion in the Se-CRN.

Face validity involves assessing the expert group’s perception of the completion of the Se-CRN Questionnaire [30]. To address this need, three open-ended questions were administered and subsequently analyzed using a qualitative approach [31]. Consideration was given to any suggestions for clarifying the items in relation to their linguistic form. Additionally, the questionnaire was revised based on the results. Each expert was asked to indicate their degree of agreement for every identified item through two distinct evaluations: essentiality (rated on a 3-point Likert scale where 1 = “not essential”; 3 = “essential”) and relevance (rated on a 4-point Likert scale where 1 = “not relevant”; 4 = “completely relevant”). These assessments were used to calculate the Content Validity Index (CVI) and the Content Validity Ratio (CVR) [32], which quantify the representativeness and essentiality of each item. In line with commonly accepted criteria, an Item-CVI (I-CVI) of ≥0.78 was considered the minimum acceptable threshold for adequate relevance when evaluated by six or more experts [33]. For the CVR, Lawshe’s critical values were adopted; therefore, a CVR ≥ 0.62 was required to determine that an item met the threshold for essentiality with a panel of ten experts [32].

### 2.2. Data Collection

The final version of the Se-CRN Questionnaire was administered through an online survey platform (SurveyMonkey©). The Se-CRN is a 5-point Likert-type scale ranging from 1 (“Not at all confident”) to 5 (“Completely confident”), with higher scores indicating greater perceived self-efficacy in performing specific professional competencies. The response anchors were designed to capture both the strength and frequency of confidence judgments, consistent with Bandura’s self-efficacy theory. Each item is introduced by the trigger question: “How confident are you in your ability to perform the following activity?”. Higher total or domain-specific scores reflect stronger perceived self-efficacy within the corresponding area of clinical research nursing practice. In addition to the self-efficacy items, the questionnaire includes a brief introductory section that collects socio-demographic and professional information (e.g., gender, age, educational background, years of experience in clinical research, institutional setting) to contextualize participants’ self-efficacy profiles.

### 2.3. Inclusion Criteria

Participation in this study was voluntary. CRNs (as the nurses caring for patients enrolled in clinical trials) [9] were eligible for inclusion if they met the following criteria: (i) Full-time employment contract; (ii) Professional experience in clinical research exceeding six months. A non-probability purposive and snowball sampling strategy was adopted, leveraging the Italian Association of Oncology Nursing network of affiliated professionals and relevant social media platforms.

### 2.4. Data Analysis

Data were analyzed using R 4.5.0 [34] and Python 3.3.1. Descriptive statistics were computed using means, standard deviations, and frequencies. Prior to factor extraction, assumptions of multivariate adequacy and factorability were evaluated. Sampling adequacy was assessed using the Kaiser–Meyer–Olkin (KMO) index, with values ≥0.80 considered meritorious for factor analysis and individual MSAs ≥ 0.70 acceptable. Bartlett’s Test of Sphericity was used to test the null hypothesis of an identity correlation matrix (α = 0.05). The number of factors to be extracted was determined using Parallel Analysis (PA) with 500 random permutations, retaining factors whose empirical eigenvalues exceeded the 95th percentile of the simulated distributions.

An Exploratory Factor Analysis (EFA) was performed using Maximum Likelihood (ML) extraction with oblimin rotation to allow for correlated latent dimensions. Factor retention was guided by convergent criteria: (i) parallel analysis, (ii) incremental improvement in model fit across competing solutions (three to six factors), (iii) interpretability/simple structure, and (iv) consistency with the a priori competency framework. Item evaluation followed an iterative inspection of the oblimin-rotated pattern matrix, communalities, and item complexity. Items were retained when primary factor loadings were ≥0.40 and secondary loadings were <0.30, or when the primary–secondary loading difference was ≥0.20. In a small number of cases with marginal cross-loadings (i.e., slightly above 0.30), items were retained if the primary loading remained clearly dominant and the content was theoretically essential to the domain. Structural validity was further evaluated using hierarchical reliability indices consistent with bifactor modelling. Internal consistency was examined using Cronbach’s alpha (α ≥ 0.70 acceptable; ≥0.90 excellent), McDonald’s omega total (ωt), omega hierarchical (ωh), Composite Reliability (CR ≥ 0.70), Signal-to-Noise Ratio (S/N > 1), and the Explained Common Variance (ECV) to evaluate the presence and strength of a general factor [35].

For inferential comparisons, three variables were dichotomized a priori based on conceptual and practice rationale within CRN literature. For setting-based comparisons, we created a dichotomous variable distinguishing Oncologic (nurses working in oncology or in both oncology and non-oncology settings) versus Non-oncologic (nurses working exclusively in non-oncology settings). Participants indicating ‘Both’ were therefore included in the Oncologic group (i.e., ‘any oncology exposure’ rule). Educational level was dichotomized as Post-basic (Master’s, postgraduate, or PhD) versus Basic (Bachelor’s degree or equivalent). Experience in setting was dichotomized as Experienced versus Novice using a ≥ 2 years threshold. The 2-year benchmark reflects the period commonly reported as necessary to transition beyond initial familiarization and to consolidate autonomous performance in complex nursing practice systems [36]. Accordingly, ≥2 years was considered an empirically defensible milestone for meaningful competency consolidation in CRN practice contexts. Associations between competence scores and participant characteristics were examined using non-parametric Mann–Whitney U tests (two-tailed α = 0.05) due to non-normality and unequal group sizes. Effect sizes were calculated as rank-biserial correlation (r) and classified according to Cohen’s criteria (≈0.10 small, ≈0.30 moderate, ≈0.50 large) [37]. Total and domain scores were calculated as the mean of their constituent items.

### 2.5. Ethical Statement

The non-interventional, minimal-risk, anonymous online survey of healthcare professionals was conducted in accordance with the principles of the Declaration of Helsinki [38] and national regulations governing research involving human participants. Participation was voluntary and anonymous, and all participants provided electronic informed consent prior to completing the questionnaire. The study protocol was reviewed and approved by the Scientific Committee of the Italian Society of Oncology Nursing under approval number 01/INT/2022. All participants gave their consent to the processing of their personal data in an anonymous and computerized format. Data were collected and analyzed in anonymous/aggregated form in accordance with data-protection requirements (GDPR).

According to applicable institutional policies for anonymous questionnaire studies, formal ethics committee was not required.

## 3. Results

A total of *n* = 183 CRNs with an average of 14.30 (SD = 8.90) years of professional experience and 7.67 (SD = 7.65) years in their current setting participated in the survey (Table 1). The majority of nurses worked in oncological settings (72.7%), while a minority worked in non-oncological settings (18.6%). In specific settings, most were in medical wards (68.9%), followed by other areas (16.4%), maternal–infant units (6.6%), surgical units (6.0%), and intensive care units (2.2%).

### 3.1. Content Validity

The initial 51-item pool was reduced to 49 items based on expert ratings. The average scale content validity index (S-CVI/Ave) was 0.96 (Supplementary File S1).

### 3.2. Structural Validity

**Figure 1:**
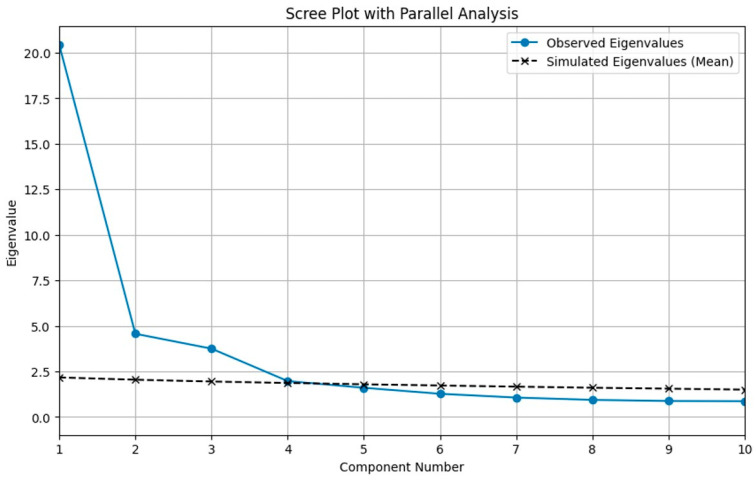
Visual inspection of the scree plot showed an elbow after the fifth factor, with a steep decline in eigenvalues from the first to the fifth component and a more gradual, plateau-like pattern thereafter. Consistent with this, the first five unrotated eigenvalues were greater than 1, whereas subsequent eigenvalues fell below this threshold, suggesting a limited contribution of additional factors to the explained variance. Parallel analysis provided a more stringent confirmation of this pattern: only the first five empirical eigenvalues exceeded the 95th percentile of the corresponding eigenvalues obtained from randomly generated data matrices, whereas all later factors were within the range expected by chance. Taken together with the a priori theoretical model, these convergent criteria supported the retention of a five-factor solution for EFA.

Parallel analysis suggested a five-factor solution (Figure 1). The four-factor model fit significantly better than the three-factor model, TRd_(52)_ = 410.56, *p* < 0.001, CD = 1.07; adding a fifth factor further improved model fit, TRd_(53)_ = 210.34, *p* < 0.001, CD = 1.03. By contrast, the six-factor solution did not provide an improvement over the five-factor model, TRd_(54)_ = 42.18, *p* = 0.882, CD = 1.01.

The collected data were suitable for EFA, χ^2^_(1176)_ = 8418.63, *p* < 0.001, KMO = 0.90 (range = 0.86–0.94).

The EFA yielded five distinct factors with interpretable loadings and minimal cross-loadings, corresponding to the theoretical domains: “Clinical Practice” (F1), “Study Management” (F2), “Human Subjects Protection” (F3), “Contributing to the Science” (F4), and “Care Coordination and Continuity” (F5) (Table 2).

### 3.3. Reliability

Internal consistency was excellent at the scale level (ωt = 0.96, (ωh) = 0.82, α = 0.96, CR = 0.96, S/N = 24.6, and ECV = 0.74), supporting a hierarchical structure with a strong general factor. Factor-level reliability indices are reported in Table 3.

### 3.4. Self-Efficacy Levels

Mean self-efficacy scores by factor and total scale are reported in Table 4.

Self-efficacy levels did not differ by clinical setting (oncology vs. non-oncology) for any factor (W range: 839–1189.5, all *p* > 0.16), as shown in Table 5. Educational level (post-basic vs basic) was associated with higher self-efficacy in Study Management (W = 1613.5, *p* = 0.002) and Contributing to the Science (W = 1631, *p* = 0.001), and on Total score (W = 1529, *p* = 0.013). The strongest differences emerged for experience in the current setting (≥2 years vs. <2 years), with higher self-efficacy across all five domains (W = 1084.5–1247, all *p* ≤ 0.010) and the Total score (W = 1247, *p* < 0.001).

## 4. Discussion

This study developed and tested the Se-CRN scale, providing the first quantitative measurement of self-efficacy across the competency domains of CRN. Whereas prior literature has primarily catalogued competencies and delineated role boundaries, our work operationalizes these domains into a psychometrically evaluated instrument anchored to the conceptual framework articulated by Bevans and Castro [20], originally derived from Hastings [21]. In a national Italian sample of CRNs, the scale showed excellent internal consistency at both total and subscale levels, and a five-factor structure that mirrors the theoretical model. 

**Figure 2:**
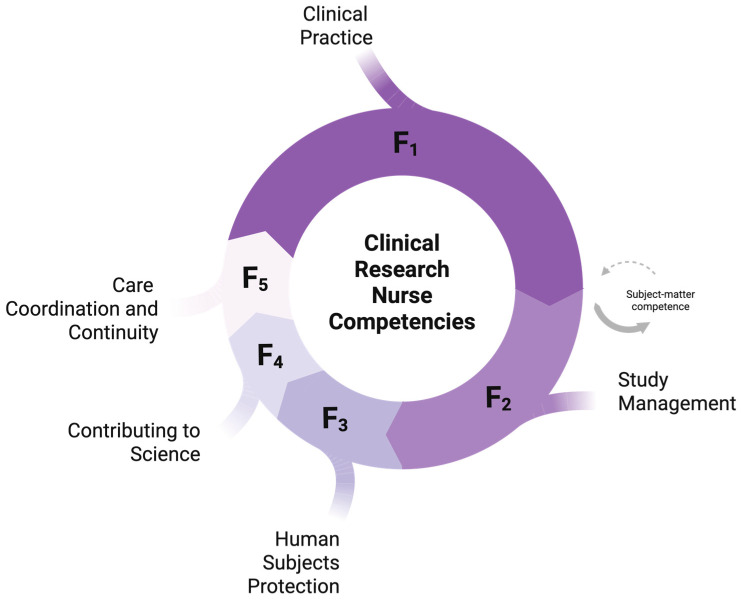
The Se-CRN scale operationalizes clinical research nurses’ self-efficacy as a multidimensional construct spanning five interlocking competency domains—Clinical Practice (F1), Study Management (F2), Human Subject Protection (F3), Contributing to the Science (F4), and Care Coordination and Continuity (F5). The relative size of each domain reflects the number of items retained for that domain. Importantly, *Clinical Practice* represents the role’s subject-matter competence: the CRN is first and foremost a clinically expert nurse who additionally holds research expertise, so research practice is necessarily grounded in specialty-specific clinical knowledge.

Exploratory analysis supported a five-factor solution: Clinical Practice, Study Management, Human Subject Protection, Contributing to the Science, and Care Coordination and Continuity (Figure 2). At the scale level, hierarchical reliability indices suggested a plausible bifactor structure with a robust general factor, supporting the concurrent reporting of total and domain scores. 

The pattern observed—higher self-efficacy in Human Subject Protection and Care Coordination and Continuity, alongside comparatively lower levels in Study Management and Contributing to the Science—aligns with prior work showing that CRN practice is often supported for protocol safety functions, whereas managerial and scholarly activities are more variably enabled by organizations [9]. Study management and scientific contribution frequently depend on access to protected time, formal authority within trial governance, and structured research method support (e.g., opportunities to contribute to protocol development) [39]. Where these conditions are limited, lower domain-specific self-efficacy may reflect constrained role enactment rather than individual deficit. Accordingly, implementation strategies that operationalize competency frameworks into local training pathways (such as structured onboarding, mentorship and competency-based benchmarking) may be particularly relevant for strengthening self-efficacy in these domains [40,41]. In parallel, widespread role ambiguity—where the CRN role is often blurred with broader “research nurse” profiles (e.g., nurses involved only in academic research setting)—and inconsistent institutional recognition may further blunt perceived capability in cross-boundary competencies such as study leadership and scholarly contribution, precisely the areas where our subscale means were lower [42,43,44]. By operationalizing the CRN taxonomy into measurable self-efficacy domains, the Se-CRN offers a way to render these historically “invisible” competencies visible and trackable.

The factor solution reflects the dual identity of the clinical research nurse as, first and foremost, a clinical nurse who practices within a research-intensive environment [45,46]. In particular, the Clinical Practice and Care Coordination and Continuity domains capture core elements of advanced clinical nursing practice, such as patient and family education, advocacy, monitoring of clinical status and adverse events, interdisciplinary care planning, and coordination of care across visits and settings. Although the wording of the items is anchored in the context of clinical research, the underlying behaviors draw on fundamental clinical nursing competencies and presuppose robust subject-matter expertise in the conditions, treatments, and trajectories of illness involved in the studies. In this sense, CRN self-efficacy, as measured by the Se-CRN, is grounded in—and cannot be disentangled from—a solid clinical foundation.

Moreover, the present study shows that, within a pool of nurses who already possess advanced clinical expertise, competence self-efficacy is not simply a function of working in oncologic versus non-oncologic contexts. Estimates did not support meaningful differences by clinical specialty. Instead, experience within the research setting emerged as the most powerful explanatory mechanism for competence. Nurses with ≥2 years of experience demonstrated materially higher levels of self-efficacy across all five factors and in the total score, with moderate non-parametric effect sizes. This pattern should not be interpreted as diminishing the centrality of clinical expertise or subject-matter knowledge; being a CRN presupposes being a clinically competent nurse in a specific field. Instead, our data suggest that, once this clinical foundation is in place, the incremental development of CRN self-efficacy is primarily driven by situated role exposure, progressive enactment, and legitimate participation in research work—rather than by disciplinary label alone [36]. In this sense, CRN competence constitutes a distinct work domain that builds on, but does not replace, specialty-based nursing practice and develops within the epistemic, regulatory, and organizational logics of clinical research itself [20]. Educational level showed greater domain-specific influence, particularly in management and scientific contributions, indicating that formal advanced education may augment learning curves preferentially in the cognitive/analytic components of the role rather than in core operational safety and coordination functions. These findings collectively underscore the importance of structured experiential pathways, supervised practice, and professional role socialization mechanisms in accelerating progression toward competent, autonomous research nursing practice [9]. Given the cross-sectional design, these associations should be interpreted cautiously: they may reflect the accumulation of mastery experiences and role socialization in research environments, but they may also be influenced by selection and retention processes (i.e., nurses who remain in CRN roles may differ from those who exit). From an implementation standpoint, the findings reinforce the value of creating early, supported exposure to research work through supervised practice and mentorship, with progressive responsibility and benchmarking against domain-specific competencies—conditions that map onto the mechanisms through which self-efficacy is strengthened (mastery experiences, vicarious learning, and social persuasion) [47].

Beyond the Italian context, the theoretical grounding of the Se-CRN in internationally established competency frameworks supports its potential applicability in other health systems where CRNs contribute to the conduct of clinical trials. At the same time, contextual interpretation is warranted. Italy does not currently maintain a national registry of CRNs; therefore, available figures are necessarily approximate. Based on expert mapping, the Italian CRN workforce is estimated at about 200–250 nurses holding formally designated research-nursing posts, largely concentrated in IRCCS and other high-volume clinical trial centers [48], and this may undercount nurses contributing to research activities in non-IRCCS hospitals under non-standardized role titles. Within this denominator, our sample of 183 respondents likely represents more than two-thirds of the target population, enabling a robust picture of current self-efficacy patterns in the national workforce. Nevertheless, international validation, particularly in systems with different regulatory structures, role delineations, and professional pathways, is essential to test the generalizability and cultural adaptability of the scale, and to expand its utility in informing education, workforce planning, and quality improvement globally [49].

### 4.1. Limitations

A primary limitation is reliance on EFA without confirmatory analysis. This decision was methodologically pragmatic given the realistically small national population of CRNs in Italy (estimated ~200–250) and the absence of a national registry, which constrained the feasibility of an adequately powered independent CFA sample. Within these constraints, EFA with parallel analysis and strong sampling adequacy criteria represents a rational first step. Additionally, the sample was predominantly oncology-based and highly educated, which may limit generalizability to other settings. Recruitment through professional networks and social media platforms may have introduced selection bias, potentially over-representing nurses who are more motivated, digitally engaged, and already interested in clinical research. As a result, the sample may not fully reflect the broader population of Italian clinical research nurses, including those with lower levels of engagement, fewer formal opportunities for research involvement, or different contractual arrangements. Also, our sample was highly educated and predominantly oncology-based. Oncology trials often involve more intensive monitoring, protocol-driven workflows, and frequent safety assessments than other areas. This context may elevate self-efficacy and could contribute to higher ‘Clinical Practice’ scores and potential ceiling effects. Therefore, mean levels and variability may differ in lower-intensity research settings (e.g., observational studies) or among nurses with less advanced education. Future studies should validate the Se-CRN in more diverse samples and examine measurement invariance across specialties and educational strata.

These factors should be considered when extrapolating the present findings to other organizational and national contexts. Importantly, the Se-CRN should be interpreted as assessing self-efficacy (perceived capability) in clinical research practice on the assumption of an existing foundation of specialty-specific clinical competence. For workforce development and educational planning, Se-CRN scores are thus best used alongside, rather than instead of, initiatives that maintain and strengthen underlying clinical expertise.

### 4.2. Implications for Practice

The Se-CRN provides a theory-grounded, measurable indicator of perceived capability in the core domains of clinical research nursing. It offers practical utility for training needs’ assessment and targeted curriculum design, particularly in areas where confidence levels are lower, such as study management and research methods. The strong general factor supports the use of a total score for screening/benchmarking. In contrast, subscale scores could guide domain-specific interventions and workforce planning decisions (e.g., skill-mix and supervision models). Because self-efficacy is intertwined with broader well-being and burnout-related processes, using the Se-CRN to detect low-confidence domains may also support early identification of psychosocial support needs and preventive strategies alongside competency development.

## 5. Conclusions

The Se-CRN provides the first validated measure of clinical research nursing self-efficacy aligned with internationally recognized competency frameworks. Its psychometric structure enables reliable assessment of perceived capability at both the general and domain-specific levels. In applied settings, the Se-CRN may support early identification of domain-specific support needs, inform preventive and targeted professional development strategies (e.g., mentorship and training in study management and scientific contribution), and guide workforce planning. Future research should confirm the factor structure, test measurement invariance, and evaluate responsiveness to education and practice change across diverse health systems.

## Figures and Tables

**Table 1:** Descriptive Characteristics of the Sample (N = 183).

Variable/Category	Value
Age (years) M (SD)	38.65 (9.18)
Experience (years) M (SD)	14.30 (8.90)
Experience in current setting (years) M (SD)	7.67 (7.65)
Sex *n* (%)	
Female	155 (84.7%)
Male	28 (15.3%)
Educational Level n (%)	
Post-basic (Master’s, postgraduate, or PhD)	150 (82.0%)
Bachelor’s degree or equivalent	33 (18.0%)
Clinical Setting n (%)	
Oncological	133 (72.7%)
Non-oncological	34 (18.6%)
Both *	16 (8.7%)

Notes. M = Mean; SD = Standard Deviation. * For dichotomized setting comparisons, the ‘Both’ subgroup was merged with the Oncologic category (any oncology exposure).

**Table 2:** Standardized factor loadings from the five–factor exploratory factor analysis.

Item (English)	Clinical Practice (F1)	Study Management (F2)	Human Subject Protection (F3)	Contributing to the Science (F4)	Care Coordination and Continuity (F5)	h^2^	u^2^	c
Facilitate research participants’ requests and concerns (Item 7)	**0.787 (0.041)**	−0.042 (0.078)	−0.109 (0.075)	−0.014 (0.079)	0.091 (0.075)	0.642	0.358	1
Provide education to study participants and their families regarding study participation. current clinical conditions. and/or disease progression (Item 10)	**0.771 (0.041)**	−0.145 (0.073)	0.016 (0.079)	0.018 (0.079)	0.055 (0.077)	0.620	0.380	1
Communicate the impact of study procedures to research participants (Item 6)	**0.716 (0.044)**	−0.004 (0.080)	−0.055 (0.077)	0.161 (0.072)	0.197 (0.070)	0.581	0.419	1
Monitor research participants and report any adverse events to a member of the research team (Item 11)	**0.616 (0.049)**	−0.093 (0.075)	0.280 (0.066)	0.118 (0.074)	−0.191 (0.070)	0.517	0.483	1
Facilitate communication within the research team (Item 34)	**0.615 (0.049)**	0.085 (0.076)	0.236 (0.068)	0.118 (0.074)	−0.072 (0.076)	0.460	0.540	1
Support research participants in defining their motivations and goals for study participation (Item 21)	**0.608 (0.050)**	0.202 (0.070)	−0.096 (0.075)	0.046 (0.078)	0.185 (0.071)	0.457	0.543	1
Collaborate with the interdisciplinary team to create and communicate a care plan that ensures safety and effective clinical research data collection (Item 2)	**0.564 (0.052)**	0.054 (0.077)	−0.050 (0.077)	0.228 (0.069)	0.282 (0.066)	0.455	0.545	1
Provide nursing expertise to the research team during study development and implementation (Item 40)	**0.540 (0.053)**	0.109 (0.075)	0.247 (0.068)	0.094 (0.075)	−0.117 (0.074)	0.387	0.613	1
Identify implications for clinical care during study development (e.g., staff skills and resources, equipment) (Item 48)	**0.537 (0.053)**	0.308 (0.065)	0.273 (0.066)	−0.105 (0.075)	−0.142 (0.073)	0.489	0.511	2
Facilitate the initial and ongoing informed consent or patient assent process (Item 20)	**0.529 (0.054)**	0.146 (0.073)	0.067 (0.077)	0.051 (0.077)	0.027 (0.079)	0.308	0.692	1
Collect participant data based on the study endpoints (Item 38)	**0.483 (0.056)**	0.152 (0.072)	0.342 (0.063)	0.130 (0.074)	−0.116 (0.074)	0.404	0.596	2
Provide advanced tutorial supervision for newly hired staff and students participating as members of the research team (Item 18)	**0.481 (0.056)**	−0.001 (0.080)	0.272 (0.066)	0.302 (0.065)	0.022 (0.079)	0.397	0.603	2
Coordinate research activities to minimize subjective risk (Item 23)	**0.472 (0.056)**	0.254 (0.067)	0.035 (0.078)	0.093 (0.075)	0.137 (0.073)	0.316	0.684	1
Provide nursing leadership within the interdisciplinary team (Item 4)	**0.451 (0.057)**	−0.088 (0.076)	0.072 (0.076)	0.347 (0.063)	0.199 (0.070)	0.377	0.623	2
Develop specific educational materials for research participants (Item 30)	**0.433 (0.058)**	0.219 (0.069)	0.163 (0.072)	0.171 (0.071)	0.029 (0.079)	0.292	0.708	1
Facilitate interdisciplinary team training on study requirements (Item 1)	**0.429 (0.059)**	0.180 (0.071)	−0.034 (0.078)	0.162 (0.072)	0.405 (0.060)	0.408	0.592	2
Identify resources needed for direct and indirect care delivery with a view to effectiveness. cost-efficiency. and quality of service (Item 44)	0.094 (0.075)	**0.762 (0.042)**	0.079 (0.076)	−0.194 (0.070)	0.147 (0.073)	0.655	0.345	1
Participate in preparing protocol-related reports for the Ethics Committee and for Sponsors/Clinical Research Organizations (CRO) (Item 32)	−0.107 (0.075)	**0.699 (0.045)**	0.083 (0.076)	0.201 (0.070)	−0.103 (0.075)	0.558	0.442	1
Support the development of study grants and budgets (Item 43)	0.116 (0.074)	**0.687 (0.046)**	−0.045 (0.078)	0.032 (0.078)	0.010 (0.080)	0.489	0.511	1
Manage potential ethical and financial conflicts of interest for oneself (Item 25)	0.012 (0.079)	**0.647 (0.048)**	−0.001 (0.080)	0.208 (0.070)	−0.252 (0.067)	0.525	0.475	1
Participate in the selection of potential research participants for eligibility (Item 28)	0.069 (0.077)	**0.640 (0.048)**	−0.049 (0.078)	0.069 (0.077)	0.329 (0.064)	0.530	0.470	2
Participate in the recruitment of research participants (Item 27)	0.061 (0.077)	**0.616 (0.049)**	−0.037 (0.078)	0.091 (0.075)	0.320 (0.064)	0.496	0.504	2
Participate in drafting the study protocol (Item 26)	0.059 (0.077)	**0.598 (0.050)**	−0.127 (0.074)	0.378 (0.061)	−0.043 (0.078)	0.522	0.478	2
Contribute to the development of CRFs (Item 35)	−0.322 (0.064)	**0.582 (0.051)**	0.304 (0.065)	0.163 (0.072)	0.111 (0.074)	0.574	0.426	3
Facilitate accurate communication with different research centres (Item 33)	−0.127 (0.074)	**0.564 (0.052)**	0.220 (0.069)	0.147 (0.073)	0.209 (0.070)	0.448	0.552	1
Supervise human resources involved in the research process (Item 45)	0.279 (0.066)	**0.517 (0.054)**	0.251 (0.067)	−0.085 (0.076)	0.023 (0.079)	0.416	0.584	1
Serve as a member of the Ethics Committee/Clinical Research Office (Item 24)	0.138 (0.073)	**0.505 (0.055)**	−0.200 (0.070)	0.348 (0.063)	−0.247 (0.068)	0.496	0.504	2
Identify research trends and participate in drafting related reports (Item 49)	0.253 (0.067)	**0.481 (0.056)**	0.137 (0.073)	0.250 (0.068)	−0.296 (0.065)	0.464	0.536	1
Record data in approved study documents (e.g., CRF, research/study database) (Item 46)	−0.060 (0.077)	**0.438 (0.058)**	0.436 (0.058)	0.142 (0.073)	−0.157 (0.072)	0.430	0.570	2
Collaborate with the interdisciplinary team in managing ethical conflicts (Item 22)	0.292 (0.065)	**0.398 (0.060)**	0.034 (0.078)	0.134 (0.073)	0.194 (0.070)	0.300	0.700	1
Comply with International Council for Harmonisation Good Clinical Practice (ICH-GCP) guidelines (Item 37)	−0.006 (0.080)	0.043 (0.078)	**0.768 (0.042)**	0.014 (0.079)	0.027 (0.079)	0.593	0.407	1
Record research data (e.g., document vital signs, administer investigational products, patient reactions) in approved documents (e.g., medical record, CRF) (Item 12)	−0.092 (0.075)	−0.068 (0.077)	**0.708 (0.045)**	0.240 (0.068)	0.049 (0.078)	0.574	0.426	1
Protect research participants’ data in compliance with regulatory requirements (Item 41)	0.164 (0.072)	0.016 (0.079)	**0.688 (0.046)**	−0.124 (0.074)	0.163 (0.072)	0.542	0.458	1
Participate in site visits and/or audits (Item 42)	0.023 (0.079)	0.131 (0.073)	**0.642 (0.048)**	0.032 (0.078)	0.171 (0.071)	0.459	0.541	1
Coordinate and facilitate the collection of research samples (Item 29)	0.301 (0.065)	0.040 (0.078)	**0.627 (0.049)**	−0.050 (0.077)	0.153 (0.072)	0.512	0.488	2
Provide direct nursing care to research participants (e.g., deliver nursing care, perform protocol interventions, collect samples) (Item 9)	0.129 (0.074)	−0.230 (0.069)	**0.622 (0.049)**	0.005 (0.080)	0.269 (0.067)	0.529	0.471	1
Facilitate processing and management (storage and transport) of research samples (Item 47)	−0.112 (0.074)	0.249 (0.068)	**0.602 (0.050)**	0.023 (0.079)	0.096 (0.075)	0.447	0.553	1
Perform quality control activities to ensure data integrity (Item 31)	0.280 (0.066)	0.148 (0.073)	**0.541 (0.053)**	0.073 (0.076)	−0.207 (0.070)	0.441	0.559	1
Facilitate scheduling and coordination of study procedures (Item 39)	0.251 (0.067)	0.118 (0.074)	**0.462 (0.057)**	−0.116 (0.074)	0.447 (0.058)	0.503	0.497	2
Participate in the setup of a study-specific database (Item 36)	−0.282 (0.066)	0.273 (0.066)	**0.353 (0.062)**	0.340 (0.063)	0.213 (0.069)	0.439	0.561	2
Generate practical questions as a result of a new study procedure or intervention (Item 15)	0.184 (0.071)	−0.011 (0.079)	−0.041 (0.078)	**0.774 (0.041)**	−0.159 (0.072)	0.660	0.340	1
Perform data analysis to contribute to the development of new ideas (Item 19)	0.088 (0.076)	0.098 (0.075)	−0.048 (0.078)	**0.756 (0.042)**	0.027 (0.079)	0.592	0.408	1
Participate in the querying and analysis of research data (Item 14)	0.094 (0.075)	0.149 (0.073)	−0.159 (0.072)	**0.696 (0.045)**	0.017 (0.079)	0.541	0.459	1
Identify appropriate questions for nursing clinical research as a result of participation in the study team (Item 17)	−0.067 (0.077)	−0.018 (0.079)	0.159 (0.072)	**0.679 (0.046)**	0.239 (0.068)	0.547	0.453	1
Collaborate with the interdisciplinary team to develop innovations in care delivery that may improve outcomes and the accuracy of research data collection (Item 16)	0.050 (0.077)	0.081 (0.076)	0.253 (0.067)	**0.620 (0.049)**	0.134 (0.073)	0.475	0.525	1
Disseminate clinical skills and best practices related to clinical research through presentations. publications. and interactions with nursing colleagues (Item 13)	0.186 (0.071)	0.151 (0.072)	0.188 (0.071)	**0.470 (0.057)**	0.151 (0.072)	0.337	0.663	1
Coordinate study participant visits (Item 3)	0.166 (0.072)	0.022 (0.079)	0.219 (0.069)	−0.013 (0.079)	**0.677 (0.046)**	0.534	0.466	1
Provide indirect nursing care (e.g., participate in unit or protocol-related visits; schedule study-related tests) in the context of research participation (Item 8)	0.159 (0.072)	0.055 (0.077)	0.202 (0.070)	0.050 (0.077)	**0.674 (0.046)**	0.525	0.475	1
Coordinate interdisciplinary meetings and activities within the study context (Item 5)	0.007 (0.080)	0.121 (0.074)	0.213 (0.069)	0.299 (0.065)	**0.629 (0.049)**	0.545	0.455	1

Note. Data are represented as Loadings (Standard Errors); loadings in bold are considered salient. h^2^ = communality; u^2^ = uniqueness; c = complexity.

**Table 3:** Summary of Reliability and Validity Measures for Each Factor.

Factor	ω	α	CR	AVE	S/N
Clinical Practice (F1)	0.923	0.932	0.892	0.455	8.259
Study Management (F2)	0.945	0.935	0.903	0.492	9.309
Human Subject Protection (F3)	0.899	0.917	0.884	0.495	7.621
Contributing to Science (F4)	0.909	0.909	0.853	0.489	5.803
Care Coordination and Continuity (F5)	0.911	0.907	0.777	0.505	3.484

Note: α = Cronbach’s alpha; ωt = McDonald’s omega total; ωh = omega hierarchical; CR = composite reliability; AVE = average variance extracted; S/N = Signal-to-Noise Ratio.

**Table 4:** Descriptive Statistics for Factor Average Scores.

Factor	M (SD)
Clinical Practice (F1)	4.01 (0.46)
Study Management (F2)	3.35 (0.66)
Human Subject Protection (F3)	4.20 (0.60)
Contributing to Science (F4)	3.37 (0.59)
Care Coordination and Continuity (F5)	4.15 (0.66)
Total	3.75 (0.60)

Notes. M = Mean; SD = Standard Deviation.

**Table 5:** Rank-biserial effect sizes (*r*) for the associations between Setting, Educational Level, and Experience in Setting with the 5 Factors and Total Competence Score.

Dichotomous Variable	Clinical Practice	Study Management	Human Subject Protection	Contributing to Science	Care Coordination & Continuity	Total
Setting (Oncologic vs. Non)	0.132 (small)	0.104 (small)	0.073 (small)	0.025 (small)	0.075 (small)	0.035 (small)
Educational Level (Post-basic vs. Basic)	0.142 (small)	**0.287** **(small)**	0.183 (small)	0.299 (small)	0.069 (small)	0.233 (small)
Experience in Setting (≥2 years vs. <2 years)	**0.337** **(moderate)**	**0.346** **(moderate)**	**0.302** **(moderate)**	**0.309** **(moderate)**	**0.242** **(small)**	**0.365** **(moderate)**

Note. Effect sizes represent rank-biserial correlations (r) derived from Mann–Whitney U tests. Effect size magnitude was interpreted according to Cohen’s benchmarks (small ≈ 0.10, moderate ≈ 0.30, large ≈ 0.50). Bold values are statistically significant. Bold values are statistically significant.

## Data Availability

The data presented in this study are available on request from the corresponding author due to ethical and privacy reasons.

## Supplementary Materials

The following supporting information can be downloaded at: https://www.mdpi.com/article/10.3390/healthcare14040551/s1, Supplementary File S1: Content Validity Procedures; Supplementary File S2: STROBE Checklist.

## Abbreviations

The following abbreviations are used in this manuscript:

AVE	Average Variance Extracted
CFA	Confirmatory Factor Analysis
CR	Composite Reliability
CRF	Case Report Form(s)
CRN	Clinical Research Nurse(s)
CRO	Clinical Research Organization(s)
CVI	Content Validity Index
CVR	Content Validity Ratio
ECV	Explained Common Variance
EFA	Exploratory Factor Analysis
GCP	Good Clinical Practice
ICH-GCP	International Council for Harmonisation—Good Clinical Practice
IRCCS	Istituto di Ricovero e Cura a Carattere Scientifico (Italian research hospital)
KMO	Kaiser–Meyer–Olkin (measure of sampling adequacy)
M	Mean
ML	Maximum Likelihood
MSA	Measure(s) of Sampling Adequacy
PA	Parallel Analysis
SD	Standard Deviation
Se-CRN	Clinical Research Nursing Competencies–Self-Efficacy (scale)
S/N	Signal-to-Noise Ratio
α	Cronbach’s alpha
c	Complexity
h^2^	Communality
u^2^	Uniqueness
ωh	Omega hierarchical
ωt	McDonald’s omega total

## References

[1] American Nurses Association, International Association of Clinical Research Nurses (2016). Clinical Research Nursing: Scope and Standards of Practice.

[2] Kalsoom Z., Victor G., Virtanen H., Sultana N. (2023). What really matters for patient safety: Correlation of nurse competence with international patient safety goals. J. Patient Saf. Risk Manag..

[3] Zaitoun R.A., Said N.B., de Tantillo L. (2023). Clinical nurse competence and its effect on patient safety culture: A systematic review. BMC Nurs..

[4] Stievano A., Caruso R., Friganović A. (2024). The Specialist Nurse in European Healthcare 2030: ESNO Congress 2024 Highlights. Healthcare.

[5] Gumber L., Agbeleye O., Inskip A., Fairbairn R., Still M., Ouma L., Lozano-Kuehne J., Bardgett M., Isaacs J.D., Wason J.M. (2024). Operational complexities in international clinical trials: A systematic review of challenges and proposed solutions. BMJ Open.

[6] Cooper E., Spilsbury K., McCaughan D., Thompson C., Butterworth T., Hanratty B. (2017). Priorities for the professional development of registered nurses in nursing homes: A Delphi study. Age Ageing.

[7] Park H., Yu S. (2019). Effective policies for eliminating nursing workforce shortages: A systematic review. Health Policy Technol..

[8] Thornton J. (2020). Clinical trials suspended in UK to prioritise COVID-19 studies and free up staff. BMJ.

[9] Bozzetti M., Guberti M., Lo Cascio A., Privitera D., Genna C., Rodelli S., Turchini L., Amatucci V., Giordano L.N., Mora V. (2025). Uncovering the Professional Landscape of Clinical Research Nursing: A Scoping Review with Data Mining Approach. Nurs. Rep..

[10] Flinkman M., Leino-Kilpi H., Numminen O., Jeon Y., Kuokkanen L., Meretoja R. (2017). Nurse Competence Scale: A systematic and psychometric review. J. Adv. Nurs..

[11] Kennedy E., Murphy G.T., Misener R.M., Alder R. (2015). Development and Psychometric Assessment of the Nursing Competence Self-Efficacy Scale. J. Nurs. Educ..

[12] Magon A., Conte G., Dellafiore F., Arrigoni C., Baroni I., Brera A.S., Avenido J., De Maria M., Stievano A., Villa G. (2023). Nursing Profession Self-Efficacy Scale-Version 2: A Stepwise Validation with Three Cross-Sectional Data Collections. Healthcare.

[13] Caruso R., Pittella F., Zaghini F., Fida R., Sili A. (2016). Development and validation of the Nursing Profession Self-Efficacy Scale. Int. Nurs. Rev..

[14] Bandura A. (1986). Social Foundations of Thought and Action: A Social Cognitive.

[15] Bandura A. (2006). Guide for Constructing Self-Efficacy Scales (Revised). Self-Effic. Beliefs Adolesc..

[16] Martín-Parrilla M.Á., Durán-Gómez N., Montanero-Fernández J., Casas-Méndez C., López-Jurado C.F., Cáceres M.C. (2025). Assessing Emotional Intelligence and Reducing Burnout in Nursing Students Through Simulation-Based Learning Experiences: An Experimental Design. Int. J. Ment. Health Nurs..

[17] Xie D., Zhu X., Zhang X., Jiang Z., Wang X., Liu T. (2024). Research on the correlation between clinical nurses’ self-efficacy, future time perspective, and occupational burnout. Front. Public Health.

[18] Anastasi G., Gravante F., Barbato P., Bambi S., Stievano A., Latina R. (2025). Moral injury and mental health outcomes in nurses: A systematic review. Nurs. Ethics.

[19] American Nurses Association (2021). The Nursing: Scope and Standards of Practice.

[20] Castro K., Bevans M., Miller-Davis C., Cusack G., Loscalzo F., Matlock A.M., Mayberry H., Tondreau L., Walsh D., Hastings C. (2011). Validating the clinical research nursing domain of practice. Oncol. Nurs. Forum.

[21] Hastings C. Putting clinical research nursing on the NIH roadmap. Proceedings of the American Academy of Nursing Annual Conference.

[22] Backman Lönn B., Hajdarevic S., Olofsson N., Hörnsten Å., Styrke J. (2022). Clarifying the role of clinical research nurses working in Sweden, using the Clinical Trial Nursing Questionnaire—Swedish version. Nurs. Open.

[23] Catania G., Poirè I., Bernardi M., Bono L., Cardinale F., Dozin B. (2012). The role of the clinical trial nurse in Italy. Eur. J. Oncol. Nurs..

[24] Ehrenberger H.E., Lillington L. (2004). Development of a measure to delineate the clinical trials nursing role. Oncol. Nurs. Forum.

[25] Milani A., Mazzocco K., Stucchi S., Magon G., Pravettoni G., Passoni C., Ciccarelli C., Tonali A., Profeta T., Saiani L. (2017). How many research nurses for how many clinical trials in an oncology setting? Definition of the Nursing Time Required by Clinical Trial-Assessment Tool (NTRCT-AT). Int. J. Nurs. Pract..

[26] Bozzetti M., Lo Cascio A., Napolitano D., Orgiana N., Mora V., Fiorini S., Petrucci G., Resente F., Baroni I., Caruso R. (2025). Measuring What Matters in Trial Operations: Development and Validation of the Clinical Trial Site Performance Measure. J. Clin. Med..

[27] Zhou Y. (2019). A Mixed Methods Model of Scale Development and Validation Analysis. Meas. Interdiscip. Res. Perspect..

[28] von Elm E., Altman D.G., Egger M., Pocock S.J., Gøtzsche P.C., Vandenbroucke J.P. (2008). The Strengthening the Reporting of Observational Studies in Epidemiology (STROBE) statement: Guidelines for reporting observational studies. J. Clin. Epidemiol..

[29] Klotz A.C., Swider B.W., Kwon S.H. (2023). Back-translation practices in organizational research: Avoiding loss in translation. J. Appl. Psychol..

[30] Connell J., Carlton J., Grundy A., Buck E.T., Keetharuth A.D., Ricketts T., Barkham M., Robotham D., Rose D., Brazier J. (2018). The importance of content and face validity in instrument development: Lessons learnt from service users when developing the Recovering Quality of Life measure (ReQoL). Qual. Life Res..

[31] Netemeyer R.G., Bearden W.O., Sharma S. (2003). Scaling Procedures: Issues and Applications.

[32] Lawshe C.H. (1975). A Quantitative Approach to Content Validity. Pers. Psychol..

[33] Polit D.F., Beck C.T. (2006). The content validity index: Are you sure you know what’s being reported? Critique and recommendations. Res. Nurs. Health.

[34] R Core Team (2023). R: A Language and Environment for Statistical Computing.

[35] Taber K.S. (2018). The Use of Cronbach’s Alpha When Developing and Reporting Research Instruments in Science Education. Res. Sci. Educ..

[36] Benner P. (1982). From novice to expert. Am. J. Nurs..

[37] Cohen J. (1992). A power primer. Psychol. Bull..

[38] World Medical Association (2013). World Medical Association Declaration of Helsinki: Ethical Principles for Medical Research Involving Human Subjects. JAMA.

[39] Morrison L., Johnston B., Cooper M. (2022). Mixed methods systematic review: Factors influencing research activity among nurses in clinical practice. J. Clin. Nurs..

[40] Jones C.T., Liu X., Hornung C.A., Fritter J., Neidecker M.V. (2024). The competency index for clinical research professionals: A potential tool for competency-based clinical research academic program evaluation. Front. Med..

[41] Bogas M., Antas J., Magalhães C., Revige M., Guerra L., Ribeiro C., Eça R.C., Nunes F., Lopes A., Costa L. (2025). Assessment of competencies of clinical research professionals and proposals to improve clinical research in Portugal. Front. Pharmacol..

[42] Napolitano D., Lo Cascio A., Bozzetti M., Guberti M. (2025). Implementing research, improving practice: Synergizing the clinical research nurse and the nurse researcher. Minerva Gastroenterol..

[43] Chen L.H., Edvardsson D., Butler A.E. (2026). Becoming, Being and Building: A Qualitative Descriptive Study of the Experiences of Clinical Research Nurses. J. Adv. Nurs..

[44] Hill G., Ellis M., Irvine L. (2022). Duality of practice in clinical research nursing. J. Res. Nurs..

[45] Kunhunny S., Salmon D. (2017). The evolving professional identity of the clinical research nurse: A qualitative exploration. J. Clin. Nurs..

[46] Lönn B.B., Hörnsten Å., Styrke J., Hajdarevic S. (2022). Transitioning to the clinical research nurse role—A qualitative descriptive study. J. Adv. Nurs..

[47] Södergård E., Juntunen J., Kuivila H.M., Tomietto M., Mikkonen K. (2025). THE Effect of Mentoring Programmes on Newly Graduated Nurses’ Retention and Turnover: An Umbrella Review. J. Adv. Nurs..

[48] Mollica G., Caruso R., Conte G., Ambrogi F., Boveri S. (2022). Analysing Researchers’ Engagement in Research Hospitals: A Pilot Study in IRCCS—Italian Research Hospitals. Healthcare.

[49] Mokkink L.B., de Vet H., Diemeer S., Eekhout I. (2023). Sample size recommendations for studies on reliability and measurement error: An online application based on simulation studies. Health Serv. Outcomes Res. Method..

