# Supplementary material for: Development and Exploratory Validation of the Clinical Research Nursing Competencies-Self-Efficacy Scale
Source: Healthcare (Basel). 2026 Feb 23;14(4):551. doi: 10.3390/healthcare14040551 (PMC12940683; doi:10.3390/healthcare14040551)
Supplement: Supplementary file 1 [file healthcare-14-00551-s001.zip › Supplementary File S2.pdf]

STROBE Statement—checklist of items that should be included in reports of observational studies.

	Item No.	Recommendation	Page No.	Relevant text from manuscript
**Title and abstract**	1	(*a*) Indicate the study's design with a commonly used term in the title or the abstract	p.1–3	Title: "Development and validation of the Clinical Research Nursing Competencies–Self-Efficacy Scale". Methods (Abstract): "A two-phase validation study was conducted using an exploratory sequential mixed-methods design…". Design (Section 2.1): "The second phase consists of a cross-sectional study for psychometric evaluation."
		(*b*) Provide in the abstract an informative and balanced summary of what was done and what was found	p.2	Abstract provides structured summary (Background/Objectives, Methods, Results, Conclusions), including sample size, key psychometric findings, and interpretation. Background/Objectives: To develop and validate the Clinical Research Nursing Competencies–Self-Efficacy (Se-CRN) scale, a theory-grounded instrument to assess perceived capability in clinical research nursing practice. Methods: A two-phase validation study was conducted using an exploratory sequential mixed-methods design between July 2022

and September 2025. The initial item pool was derived from an established competency taxonomy and refined through expert review for face and content validity. The final version of the Se-CRN was administered online to Clinical Research Nurses working with patients enrolled in clinical trials in Italy. Structural validity was examined using exploratory factor analysis with parallel analysis, and reliability was assessed through internal consistency and hierarchical indices. Group differences in self-efficacy were examined across clinical settings, educational levels, and research experience. Results: A total of 183 nurses participated. The data supported a five-factor solution reflecting core dimensions of clinical research nursing (Clinical Practice, Study Management, Human Subjects Protection, Contributing to the Science, and Care Coordination and Continuity). Reliability was excellent at the scale level and strong across domains. No differences in perceived capability were observed between oncological and non-

				oncological settings. Higher self-efficacy was consistently associated with greater experience in the research setting and, to a lesser extent, with advanced education. Conclusions: The Se-CRN is the first validated self-efficacy instrument that captures the full scope of clinical research nursing practice. It provides a practical measure to support training needs assessment, curriculum development, and workforce planning. Further research should confirm the factor structure and examine responsiveness to professional development across diverse settings.
**Introduction**				
Background/rationale	2	Explain the scientific background and rationale for the investigation being reported	p.2–3	Thus far, no available tool has been designed or psychometrically tested to capture self-efficacy as a theory-grounded proxy of CRN competence across the full scope of research activities [9]. Existing measures typically assess generic professional confidence or focus on isolated task domains, providing limited value for evaluating readiness to manage increasingly complex trials or for guiding

				targeted professional development. Without robust self-efficacy metrics, educational interventions cannot be rigorously assessed, competency gaps cannot be systematically monitored, and the contribution of CRNs to research quality and participant safety remains under-operationalized. For these reasons, this study reports the development and psychometric evaluation of the Clinical Research Nursing Competencies–Self-Efficacy (Se-CRN) scale, theoretically grounded in the Bevans–Castro taxonomy [17].
Objectives	3	State specific objectives, including any prespecified hypotheses	p.3	In this study, we aim to develop a theory-grounded, practice-relevant metric for evaluating CRN self-efficacy in the context of increasingly complex clinical trials. The secondary objective was to identify and describe CRNs' competencies in Italy.
**Methods**				
Study design	4	Present key elements of study design early in the paper	p.3	A validation study was conducted from July 2022 to September 2025, comprising two sequential phases within an exploratory sequential mixed methods design [23]. The first phase involves scale

				derivation, including construct definition and item generation, expert consultation and item revision, quantitative content validity analysis, and pilot testing for comprehensibility. The second phase consists of a cross-sectional study for psychometric evaluation.
Setting	5	Describe the setting, locations, and relevant dates, including periods of recruitment, exposure, follow-up, and data collection	p.2–4	Abstract: "...between July 2022 and September 2025... administered online to Clinical Research Nurses... in Italy."<br>A validation study was conducted from July 2022 to September 2025, comprising two sequential phases within an exploratory sequential mixed methods design [23]. The first phase involves scale derivation, including construct definition and item generation, expert consultation and item revision, quantitative content validity analysis, and pilot testing for comprehensibility. The second phase consists of a cross-sectional study for psychometric evaluation.<br>Data collection: The final version of the Se-CRN Questionnaire was administered through an online survey platform (SurveyMonkey©). The Se-CRN is a 5-point Likert-type scale ranging

				from 1 (“Not at all confident”) to 5 (“Completely confident”), with higher scores indicating greater perceived self-efficacy in performing specific professional competencies. The response anchors were designed to capture both the strength and frequency of confidence judgments, consistent with Bandura’s self-efficacy theory. Each item is introduced by the trigger question: “How confident are you in your ability to perform the following activity?”. Higher total or domain-specific scores reflect stronger perceived self-efficacy within the corresponding area of clinical research nursing practice. In addition to the self-efficacy items, the questionnaire includes a brief introductory section that collects socio-demographic and professional information (e.g., gender, age, educational background, years of experience in clinical research, institutional setting) to contextualize participants’ self-efficacy profiles.
Participants	6	(*a*) *Cohort study*—Give the eligibility criteria, and the sources and methods of selection of participants. Describe methods of follow-up<br>*Case-control study*—Give the eligibility criteria, and the sources and methods of case ascertainment and control selection. Give the rationale for the choice of cases and controls	p.4	Participation in the study was voluntary. CRNs (as the nurses caring for patients enrolled in clinical trials) [9] were eligible for

		Cross-sectional study—Give the eligibility criteria, and the sources and methods of selection of participants		inclusion if they met the following criteria: i) Full-time employment contract; ii) Professional experience in clinical research exceeding six months. A non-probability purposive and snowball sampling strategy was adopted, leveraging the [blinded for peer review] network of affiliated professionals and relevant social media platforms.
		(*b*) *Cohort study*—For matched studies, give matching criteria and number of exposed and unexposed<br>*Case-control study*—For matched studies, give matching criteria and the number of controls per case	N/A	Not applicable (no matching was performed).
Variables	7	Clearly define all outcomes, exposures, predictors, potential confounders, and effect modifiers. Give diagnostic criteria, if applicable	p.4–5	Outcome: Se-CRN total and domain self-efficacy scores.<br>The final version of the Se-CRN Questionnaire was administered through an online survey platform (SurveyMonkey©). The Se-CRN is a 5-point Likert-type scale ranging from 1 (“Not at all confident”) to 5 (“Completely confident”), with higher scores indicating greater perceived self-efficacy in performing specific professional competencies. The response anchors were designed to capture both the strength and frequency of confidence judgments, consistent with Bandura’s self-efficacy theory.

Each item is introduced by the trigger question: “How confident are you in your ability to perform the following activity?”. Higher total or domain-specific scores reflect stronger perceived self-efficacy within the corresponding area of clinical research nursing practice. In addition to the self-efficacy items, the questionnaire includes a brief introductory section that collects socio-demographic and professional information (e.g., gender, age, educational background, years of experience in clinical research, institutional setting) to contextualize participants’ self-efficacy profiles. For inferential comparisons, three variables were dichotomized a priori based on conceptual and practice rationale within CRN literature. Clinical setting was dichotomized as Oncologic (oncology or mixed oncology units) versus Non-oncologic. Educational level was dichotomized as Post-basic (Master’s, postgraduate, or PhD) versus Basic (Bachelor’s degree or equivalent). Experience in setting was dichotomized

				as Experienced versus Novice using a ≥2 years threshold. The 2-year benchmark reflects the period commonly reported as necessary to transition beyond initial familiarization and to consolidate autonomous performance in complex nursing practice systems [28]. Accordingly, ≥2 years was considered an empirically defensible milestone for meaningful competency consolidation in CRN practice contexts. Associations between competence scores and participant characteristics were examined using non-parametric Mann–Whitney U tests (two-tailed α = 0.05) due to non-normality and unequal group sizes. Effect sizes were calculated as rank-biserial correlation (r) and classified according to Cohen's criteria (≈0.10 small, ≈0.30 moderate, ≈0.50 large)[29]. Total and domain scores were calculated as the mean of their constituent items.
Data sources/ measurement	8*	For each variable of interest, give sources of data and details of methods of assessment (measurement). Describe comparability of assessment methods if there is more than one group	p.4–5	Instrument development and validation procedures:<br>Face validity involves assessing the expert group's perception of the completion of the Se-CRN

Questionnaire (Bolarinwa, 2015). To address this need, three open-ended questions were administered and subsequently analyzed using a qualitative approach (Netemeyer et al., 2003). Consideration was given to any suggestions for clarifying the items in relation to their linguistic form. Additionally, the questionnaire was revised based on the results. Each expert was asked to indicate their degree of agreement for every identified item through two distinct evaluations: essentiality (rated on a 3-point Likert scale where 1 = “not essential”; 3 = “essential”) and relevance (rated on a 4-point Likert scale where 1 = “not relevant”; 4 = “completely relevant”). These assessments were used to calculate the Content Validity Index (CVI) and the Content Validity Ratio (CVR) [24], which quantify the representativeness and essentiality of each item. In line with commonly accepted criteria, an Item-CVI (I-CVI) of ≥ 0.78 was considered the minimum acceptable threshold for adequate relevance when evaluated by six or more experts [25]. For the CVR,

Lawshe's critical values were adopted; therefore, a CVR ≥ 0.62 was required to determine that an item met the threshold for essentiality with a panel of ten experts [24].

The final version of the Se-CRN Questionnaire was administered through an online survey platform (SurveyMonkey©). The Se-CRN is a 5-point Likert-type scale ranging from 1 ("Not at all confident") to 5 ("Completely confident"), with higher scores indicating greater perceived self-efficacy in performing specific professional competencies. The response anchors were designed to capture both the strength and frequency of confidence judgments, consistent with Bandura's self-efficacy theory. Each item is introduced by the trigger question: "How confident are you in your ability to perform the following activity?". Higher total or domain-specific scores reflect stronger perceived self-efficacy within the corresponding area of clinical research nursing practice. In addition to the self-efficacy items, the questionnaire includes a brief introductory section

				that collects socio-demographic and professional information (e.g., gender, age, educational background, years of experience in clinical research, institutional setting) to contextualize participants' self-efficacy profiles.
Bias	9	Describe any efforts to address potential sources of bias	p.4; p.15–16	Sampling: "A non-probability purposive and snowball sampling strategy was adopted…". Participation in the study was voluntary. CRNs (as the nurses caring for patients enrolled in clinical trials) [9] were eligible for inclusion if they met the following criteria: i) Full-time employment contract; ii) Professional experience in clinical research exceeding six months. A non-probability purposive and snowball sampling strategy was adopted, leveraging the [blinded for peer review] network of affiliated professionals and relevant social media platforms.<br>Limitations: Recruitment through professional networks and social media platforms may have introduced selection bias, potentially over-representing nurses who are more motivated, digitally engaged, and already interested in

				clinical research.
Study size	10	Explain how the study size was arrived at	p.5; p.15–16	Sample size was determined pragmatically by recruitment within the eligible network; a formal a priori sample size calculation is not reported.<br>Results: A total of n=183 CRNs with an average of 14.30 (SD=8.90) years of professional experience and 7.67 (SD=7.65) years in their current setting participated in the survey. The majority of nurses worked in oncological settings (72.7%) while a minority worked in non-oncological settings (18.6%). In specific settings, most were in medical wards (68.9%), followed by other areas (16.4%), maternal–infant units (6.6%), surgical units (6.0%), and intensive care units (2.2%).<br>Discussion: "...small national population of CRNs in Italy (estimated ~200–250)... our sample of 183 respondents likely represents more than two-thirds of the target population."

Continued on next page

Quantitative variables	11	Explain how quantitative variables were handled in the analyses. If applicable, describe which groupings were chosen and why	p.5	For inferential comparisons, three variables were dichotomized a priori based on conceptual and practice rationale within CRN literature. Clinical setting was dichotomized as Oncologic (oncology or mixed oncology units) versus Non-oncologic. Educational level was dichotomized as Post-basic (Master's, postgraduate, or PhD) versus Basic (Bachelor's degree or equivalent). Experience in setting was dichotomized as Experienced versus Novice using a ≥2 years threshold. The 2-year benchmark reflects the period commonly reported as necessary to transition beyond initial familiarization and to consolidate autonomous performance in complex nursing practice systems [28]. Accordingly, ≥2 years was considered an empirically defensible milestone for meaningful competency consolidation in CRN practice contexts. Associations between competence scores and participant characteristics were examined using non-parametric Mann–Whitney U tests (two-tailed $\alpha = 0.05$) due to non-normality and

				unequal group sizes. Effect sizes were calculated as rank-biserial correlation (r) and classified according to Cohen's criteria (≈0.10 small, ≈0.30 moderate, ≈0.50 large)[29]. Total and domain scores were calculated as the mean of their constituent items.
Statistical methods	12	(*a*) Describe all statistical methods, including those used to control for confounding	p.4–5	Data were analyzed using R 4.5.0 [26] and Python 3.3.1. Descriptive statistics were computed using means, standard deviations, and frequencies. Prior to factor extraction, assumptions of multivariate adequacy and factorability were evaluated. Sampling adequacy was assessed using the Kaiser–Meyer–Olkin (KMO) index, with values ≥0.80 considered meritorious for factor analysis and individual MSAs ≥0.70 acceptable. Bartlett's Test of Sphericity was used to test the null hypothesis of an identity correlation matrix ($\alpha = 0.05$). The number of factors to be extracted was determined using Parallel Analysis (PA) with 500 random permutations, retaining factors whose empirical eigenvalues exceeded the 95th percentile of the simulated distributions.

			An Exploratory Factor Analysis (EFA) was performed using Maximum Likelihood (ML) extraction with oblimin rotation to allow for correlated latent dimensions. Items were retained when primary factor loadings were ≥0.40, and cross-loadings were <0.30. Structural validity was further evaluated using hierarchical reliability indices consistent with bifactor modelling. Internal consistency was examined using Cronbach's alpha (α ≥0.70 acceptable; ≥0.90 excellent), McDonald's omega total (ωt), omega hierarchical (ωh), Composite Reliability (CR ≥0.70), Signal-to-Noise Ratio (S/N > 1), and the Explained Common Variance (ECV) to evaluate the presence and strength of a general factor [27].
	(*b*) Describe any methods used to examine subgroups and interactions	p.5; p.7–8	Subgroup comparisons were planned across setting, educational level, and experience.<br>For inferential comparisons, three variables were dichotomized a priori based on conceptual and practice rationale within CRN literature. Clinical setting was dichotomized

as Oncologic (oncology or mixed oncology units) versus Non-oncologic. Educational level was dichotomized as Post-basic (Master's, postgraduate, or PhD) versus Basic (Bachelor's degree or equivalent). Experience in setting was dichotomized as Experienced versus Novice using a ≥2 years threshold. The 2-year benchmark reflects the period commonly reported as necessary to transition beyond initial familiarization and to consolidate autonomous performance in complex nursing practice systems [28]. Accordingly, ≥2 years was considered an empirically defensible milestone for meaningful competency consolidation in CRN practice contexts. Associations between competence scores and participant characteristics were examined using non-parametric Mann–Whitney U tests (two-tailed $\alpha = 0.05$) due to non-normality and unequal group sizes. Effect sizes were calculated as rank-biserial correlation (r) and classified according to Cohen's criteria (≈0.10 small, ≈0.30 moderate, ≈0.50 large)[29]. Total and domain

			scores were calculated as the mean of their constituent items. Self-efficacy levels did not differ by clinical setting (oncology vs non-oncology) for any factor (W range: 839–1189.5, all p > 0.16; effect sizes small), as shown in Table 5. Educational level (post-basic vs basic) was associated with higher self-efficacy in Study Management (W = 1613.5, p = 0.002) and Contributing to the Science (W = 1631, p = 0.001), and a small-to-moderate effect on Total score (W = 1529, p = 0.013). The strongest differences emerged for experience in the current setting (≥2 years vs <2 years), with higher self-efficacy across all five domains (W = 1084.5–1247, all p ≤ 0.010) and the Total score (W = 1247, p < 0.001). Effect sizes were moderate for four domains and the Total score (r = 0.302–0.365).
	(*c*) Explain how missing data were addressed	Not reported	Handling of missing data was not explicitly described in the manuscript.
	(*d*) *Cohort study*—If applicable, explain how loss to follow-up was addressed<br>*Case-control study*—If applicable, explain how matching of cases and controls was addressed<br>*Cross-sectional study*—If applicable, describe analytical methods taking account of sampling strategy	p.4–5	Sampling strategy: "A non-probability purposive and snowball sampling strategy was adopted…". Participation in the study was voluntary. CRNs (as the nurses

				caring for patients enrolled in clinical trials) [9] were eligible for inclusion if they met the following criteria: i) Full-time employment contract; ii) Professional experience in clinical research exceeding six months. A non-probability purposive and snowball sampling strategy was adopted, leveraging the [blinded for peer review] network of affiliated professionals and relevant social media platforms. No weighting or other adjustments for the sampling strategy are described.
		(*e*) Describe any sensitivity analyses	Not reported	No sensitivity analyses were described.
**Results**				
Participants	13*	(a) Report numbers of individuals at each stage of study—eg numbers potentially eligible, examined for eligibility, confirmed eligible, included in the study, completing follow-up, and analysed	p.5	A total of n=183 CRNs with an average of 14.30 (SD=8.90) years of professional experience and 7.67 (SD=7.65) years in their current setting participated in the survey. The majority of nurses worked in oncological settings (72.7%) while a minority worked in non-oncological settings (18.6%). In specific settings, most were in medical wards (68.9%), followed by other areas (16.4%), maternal–

				infant units (6.6%), surgical units (6.0%), and intensive care units (2.2%).
		(b) Give reasons for non-participation at each stage	Not reported	Numbers potentially eligible and reasons for non-participation were not reported.
		(c) Consider use of a flow diagram	Not included	No participant flow diagram was included.
Descriptive data	14*	(a) Give characteristics of study participants (eg demographic, clinical, social) and information on exposures and potential confounders	p.5–6 (Table 1)	Participant characteristics are presented in Table 1.<br>A total of n=183 CRNs with an average of 14.30 (SD=8.90) years of professional experience and 7.67 (SD=7.65) years in their current setting participated in the survey. The majority of nurses worked in oncological settings (72.7%) while a minority worked in non-oncological settings (18.6%). In specific settings, most were in medical wards (68.9%), followed by other areas (16.4%), maternal–infant units (6.6%), surgical units (6.0%), and intensive care units (2.2%).
		(b) Indicate number of participants with missing data for each variable of interest	Not reported	The number of participants with missing data for each variable was not reported.
		(c) *Cohort study*—Summarise follow-up time (eg, average and total amount)	N/A	Not applicable (cross-sectional study).
Outcome data	15*	*Cohort study*—Report numbers of outcome events or summary measures over time	N/A	Not applicable (cross-sectional

			study).
	Case-control study—Report numbers in each exposure category, or summary measures of exposure	N/A	Not applicable (cross-sectional study).
	Cross-sectional study—Report numbers of outcome events or summary measures	p.6–8 (Tables 2–5; Figures 1–2)	The initial 51-item pool was reduced to 49 items based on expert ratings. The average scale content validity index (S-CVI/Ave) was 0.96 (Supplementary File S1). Parallel analysis suggested a five-factor solution. The four-factor model fit significantly better than the three-factor model, TRd(52) = 410.56, $p < 0.001$, CD = 1.07; adding a fifth factor further improved model fit, TRd(53) = 210.34, $p < 0.001$, CD = 1.03. By contrast, the six-factor solution did not provide an improvement over the five-factor model, TRd(54) = 42.18, $p = 0.882$, CD = 1.01. Internal consistency was excellent at the scale level ($\omega t = 0.96$, (ωh) = 0.82, $\alpha = 0.96$, CR = 0.96, S/N = 24.6, and ECV = 0.74), supporting a hierarchical structure with a strong general factor. Factor-level reliability indices are reported in Table 3.<br>Self-efficacy levels did not differ by clinical setting (oncology vs non-oncology) for any factor (W range: 839–1189.5, all $p > 0.16$;

				effect sizes small), as shown in Table 5. Educational level (post-basic vs basic) was associated with higher self-efficacy in Study Management (W = 1613.5, p = 0.002) and Contributing to the Science (W = 1631, p = 0.001), and a small-to-moderate effect on Total score (W = 1529, p = 0.013). The strongest differences emerged for experience in the current setting (≥2 years vs <2 years), with higher self-efficacy across all five domains (W = 1084.5–1247, all p ≤ 0.010) and the Total score (W = 1247, p < 0.001). Effect sizes were moderate for four domains and the Total score (r = 0.302–0.365).
Main results	16	(*a*) Give unadjusted estimates and, if applicable, confounder-adjusted estimates and their precision (eg, 95% confidence interval). Make clear which confounders were adjusted for and why they were included	p.7–8 (Table 5)	Self-efficacy levels did not differ by clinical setting (oncology vs non-oncology) for any factor (W range: 839–1189.5, all p > 0.16; effect sizes small), as shown in Table 5. Educational level (post-basic vs basic) was associated with higher self-efficacy in Study Management (W = 1613.5, p = 0.002) and Contributing to the Science (W = 1631, p = 0.001), and a small-to-moderate effect on Total score (W = 1529, p = 0.013). The strongest differences emerged for

			experience in the current setting (≥2 years vs <2 years), with higher self-efficacy across all five domains (W = 1084.5–1247, all $p \leq 0.010$) and the Total score (W = 1247, $p < 0.001$). Effect sizes were moderate for four domains and the Total score (r = 0.302–0.365).
	(*b*) Report category boundaries when continuous variables were categorized	p.5	Continuous variables were categorized using predefined boundaries. For inferential comparisons, three variables were dichotomized a priori based on conceptual and practice rationale within CRN literature. Clinical setting was dichotomized as Oncologic (oncology or mixed oncology units) versus Non-oncologic. Educational level was dichotomized as Post-basic (Master's, postgraduate, or PhD) versus Basic (Bachelor's degree or equivalent). Experience in setting was dichotomized as Experienced versus Novice using a ≥2 years threshold. The 2-year benchmark reflects the period commonly reported as necessary to transition beyond initial familiarization and to consolidate autonomous performance in

			complex nursing practice systems [28]. Accordingly, ≥2 years was considered an empirically defensible milestone for meaningful competency consolidation in CRN practice contexts. Associations between competence scores and participant characteristics were examined using non-parametric Mann–Whitney U tests (two-tailed $\alpha = 0.05$) due to non-normality and unequal group sizes. Effect sizes were calculated as rank-biserial correlation (r) and classified according to Cohen's criteria (≈0.10 small, ≈0.30 moderate, ≈0.50 large)[29]. Total and domain scores were calculated as the mean of their constituent items.
	(*c*) If relevant, consider translating estimates of relative risk into absolute risk for a meaningful time period	N/A	Not applicable (no relative risks were estimated).

Continued on next page

Other analyses	17	Report other analyses done—eg analyses of subgroups and interactions, and sensitivity analyses	p.5; p.7–8	Other analyses consisted of subgroup comparisons by setting, education, and experience (no sensitivity analyses reported). Self-efficacy levels did not differ by clinical setting (oncology vs non-oncology) for any factor (W range: 839–1189.5, all $p > 0.16$; effect sizes small), as shown in Table 5. Educational level (post-basic vs basic) was associated with higher self-efficacy in Study Management ($W = 1613.5$, $p = 0.002$) and Contributing to the Science ($W = 1631$, $p = 0.001$), and a small-to-moderate effect on Total score ($W = 1529$, $p = 0.013$). The strongest differences emerged for experience in the current setting (≥2 years vs <2 years), with higher self-efficacy across all five domains (W = 1084.5–1247, all $p \leq 0.010$) and the Total score ($W = 1247$, $p < 0.001$). Effect sizes were moderate for four domains and the Total score (r = 0.302–0.365).
**Discussion**				
Key results	18	Summarise key results with reference to study objectives	p.12–16	Discussion summarises key results in line with objectives. This study developed and tested the Se-CRN scale, providing the

				first quantitative measurement of self-efficacy across the competency domains of CRN. Whereas prior literature has primarily catalogued competencies and delineated role boundaries, our work operationalizes these domains into a psychometrically evaluated instrument anchored to the conceptual framework articulated by Bevans and Castro [17], originally derived from Hastings [18]. In a national Italian sample of CRNs, the scale showed excellent internal consistency at both total and subscale levels, and a five-factor structure that mirrors the theoretical model.
Limitations	19	Discuss limitations of the study, taking into account sources of potential bias or imprecision. Discuss both direction and magnitude of any potential bias	p.15–16	A primary limitation is reliance on EFA without confirmatory analysis. This decision was methodologically pragmatic given the realistically small national population of CRNs in Italy (estimated ~200–250) and the absence of a national registry, which constrained the feasibility of an adequately powered independent CFA sample. Within these constraints, EFA with parallel analysis and strong sampling adequacy criteria represents a rational first step. Additionally, the

sample was predominantly oncology-based and highly educated, which may limit generalizability to other settings. Recruitment through professional networks and social media platforms may have introduced selection bias, potentially over-representing nurses who are more motivated, digitally engaged, and already interested in clinical research. As a result, the sample may not fully reflect the broader population of Italian clinical research nurses, including those with lower levels of engagement, fewer formal opportunities for research involvement, or different contractual arrangements. These factors should be considered when extrapolating the present findings to other organizational and national contexts. Importantly, the Se-CRN should be interpreted as assessing self-efficacy (perceived capability) in clinical research practice on the assumption of an existing foundation of specialty-specific clinical competence. For workforce development and educational planning, Se-CRN scores are thus best used alongside, rather than

				instead of, initiatives that maintain and strengthen underlying clinical expertise.<br>Recruitment through professional networks and social media platforms may have introduced selection bias, potentially over-representing nurses who are more motivated, digitally engaged, and already interested in clinical research.
Interpretation	20	Give a cautious overall interpretation of results considering objectives, limitations, multiplicity of analyses, results from similar studies, and other relevant evidence	p.12–16	Interpretation in context of existing evidence:<br>The pattern we observed—higher self-efficacy in Human Subjects Protection and Care Coordination and Continuity, alongside comparatively lower levels in Study Management and Contributing to Science—maps closely onto the international literature synthesized in a recent scoping review [9]. The review documents persistent structural and educational constraints (e.g., limited protected time, uneven access to training, and fragile career pathways) that are most likely to depress confidence in managerial/administrative tasks and in research generation/translation activities, while routine GCP-anchored functions remain

				comparatively well supported in practice. In parallel, widespread role ambiguity (often conflating the CRN with broader "research nurse" functions) and inconsistent institutional recognition may further blunt perceived capability in cross-boundary competencies such as study leadership and scholarly contribution—precisely the areas where our subscale means were lower [30]. By operationalizing the CRN taxonomy into measurable self-efficacy domains, the Se-CRN offers a way to render these historically "invisible" competencies visible and trackable.
Generalisability	21	Discuss the generalisability (external validity) of the study results	p.15–16	Beyond the Italian context, the theoretical grounding of the Se-CRN in internationally established competency frameworks supports its potential applicability in other health systems where CRNs contribute to the conduct of clinical trials. At the same time, contextual interpretation is warranted. Italy does not currently maintain a national registry of CRNs, but expert estimates suggest a workforce of approximately 200–250 nurses formally engaged in research activities [33]. Within this

				denominator, our sample of 183 respondents likely represents more than two-thirds of the target population, enabling a robust picture of current self-efficacy patterns in the national workforce. Nevertheless, international validation, particularly in systems with different regulatory structures, role delineations, and professional pathways, is essential to test the generalisability and cultural adaptability of the scale, and to expand its utility in informing education, workforce planning, and quality improvement globally [34].
**Other information**				
Funding	22	Give the source of funding and the role of the funders for the present study and, if applicable, for the original study on which the present article is based	p.17	Funding: This research received no external funding.

*Give information separately for cases and controls in case–control studies and, if applicable, for exposed and unexposed groups in cohort and cross-sectional studies.

Note: An Explanation and Elaboration article discusses each checklist item and gives methodological background and published examples of transparent reporting. The STROBE checklist is best used in conjunction with this article (freely available on the Web sites of PLoS Medicine at http://www.plosmedicine.org/, Annals of Internal Medicine at http://www.annals.org/, and Epidemiology at http://www.epidem.com/). Information on the STROBE Initiative is available at www.strobe-statement.org.
