# Supplementary material for: Development and Exploratory Validation of the Clinical Research Nursing Competencies-Self-Efficacy Scale
Source: Healthcare (Basel). 2026 Feb 23;14(4):551. doi: 10.3390/healthcare14040551 (PMC12940683; doi:10.3390/healthcare14040551)
Supplement: Supplementary file 1 [file healthcare-14-00551-s001.zip › Supplementary File S1.pdf]

Supplementary File 1. Content validity procedures

First evaluation (essentiality).

Items	Ne	N/2	CVR	Consensus CVR > 0.60
Facilitates educating the interdisciplinary team about the study requirements (Item 1)	13	7.5	0.733	In
Works with the interdisciplinary team to create and communicate a plan of care that ensures the safe and effective collection of clinical research data (Item 2)	15	7.5	1.000	In
Coordinates study visits for research participants (Item 3)	12	7.5	0.600	In
Provides nursing leadership within the interdisciplinary team (Item 4)	14	7.5	0.866	In
Coordinates interdisciplinary meetings and activities within the context of the study (Item 5)	13	7.5	0.733	In
Coordinates referrals of patients to interdisciplinary services outside the research team	11	7.5	0.466	Out
Communicates the impact of study procedures to research participants (Item 6)	15	7.5	1.000	In

Facilitates research participants' questions and concerns (Item 7)	14	7.5	0.866	In
Provides indirect nursing care (e.g.. participating in clinical. unit. and/or protocol rounds; planning study-related tests) in the context of research participation (Item 8)	14	7.5	0.866	In
Provides direct nursing care to research participants (e.g.. interacting with participants to deliver nursing care. carrying out research-related interventions. collecting specimens) (Item 9)	14	7.5	0.866	In
Provides education to research participants and their families about study participation. the patient's current clinical condition. and/or the course of the disease (Item 10)	15	7.5	1.000	In
Monitors research participants and reports potential adverse events to a member of the research team (Item 11)	15	7.5	1.000	In
Records research data (e.g.. documenting vital signs. administering investigational products. participant responses) in approved source documents (e.g.. the medical record. data collection forms) (Item 12)	15	7.5	1.000	In
Shares clinical expertise and best practices related to clinical research through presentations. publications. and interactions with nursing colleagues (Item 13)	14	7.5	0.866	In
Takes part in querying and analyzing research data (Item 14)	14	7.5	0.866	In
Generates practice-focused questions as a result of new study procedures or interventions (Item 15)	14	7.5	0.866	In

Collaborates with the interdisciplinary team to develop innovations in care delivery that have the potential to improve outcomes and the accuracy of research data collection (Item 16)	14	7.5	0.866	In
Identifies questions suitable for clinical nursing research that arise from participation in the study team (Item 17)	13	7.5	0.733	In
Provides advanced mentoring and supervision for newly hired staff and for students participating as members of the research team (Item 18)	14	7.5	0.866	In
Performs secondary analyses of data to contribute to the development of new ideas (Item 19)	12	7.5	0.600	In
Serves as a resource for new researchers	11	7.5	0.466	Out
Facilitates the initial and ongoing informed consent or assent process (Item 20)	14	7.5	0.866	In
Supports research participants in defining their reasons and goals for taking part in a study (Item 21)	15	7.5	1.000	In
Works with the interdisciplinary team to address ethical conflicts (Item 22)	15	7.5	1.000	In
Coordinates research activities to minimize risk to participants (Item 23)	15	7.5	1.000	In

Serves as a member of the ethics committee or clinical research office (e.g.. institutional review board) (Item 24)	13	7.5	0.733	In
Manages potential ethical and financial conflicts of interest for oneself (Item 25)	14	7.5	0.866	In
Participates in study development (Item 26)	12	7.5	0.600	In
Participates in recruiting research participants (Item 27)	13	7.5	0.733	In
Participates in screening potential research participants for eligibility (Item 28)	13	7.5	0.733	In
Coordinates and facilitates the collection of research specimens (Item 29)	15	7.5	1.000	In
Develops study-specific materials for research participant education (Item 30)	14	7.5	0.866	In
Performs quality assurance activities to ensure data integrity (Item 31)	15	7.5	1.000	In
Participates in preparing protocol-related reports for ethics committees and sponsors/clinical research organizations (Item 32)	12	7.5	0.600	In

Facilitates accurate communication between research sites (Item 33)	13	7.5	0.733	In
Facilitates communication within the research team (Item 34)	15	7.5	1.000	In
Contributes to the development of case report forms (Item 35)	13	7.5	0.733	In
Participates in setting up a study-specific database (Item 36)	13	7.5	0.733	In
Adheres to International Conference on Harmonisation guidelines for Good Clinical Practice (Item 37)	15	7.5	1.000	In
Collects data on research participants based on the study endpoints (Item 38)	14	7.5	0.866	In
Facilitates scheduling and coordination of study procedures (Item 39)	14	7.5	0.866	In
Provides nursing expertise to the research team during study development and implementation (Item 40)	13	7.5	0.733	In
Safeguards research participant data in line with regulatory requirements (Item 41)	14	7.5	0.866	In

Takes part in site visits and/or audits (Item 42)	15	7.5	1	In
Supports the development of study grants and budgets (Item 43)	13	7.5	0.733	In
Identifies the resources needed to provide direct and indirect care. with attention to efficiency. cost-effectiveness. and quality of services delivered (Item 44)	14	7.5	0.866	In
Oversees the human resources involved in the research process (Item 45)	12	7.5	0.600	In
Records data on approved study documents (e.g.. case report forms. research or study databases) (Item 46)	14	7.5	0.866	In
Facilitates the processing and handling (storage and transport) of research specimens (Item 47)	15	7.5	1.000	In
Identifies implications for clinical care during study development (e.g.. staff competencies and resources. equipment) (Item 48)	14	7.5	0.866	In
Identifies research trends and contributes to the preparation of related reports (Item 49)	14	7.5	0.866	In

Second Evaluation (Relevance)

Items	RATE ³3	I-CVIs
Facilitates educating the interdisciplinary team about the study requirements (Item 1)	13	0.866
Works with the interdisciplinary team to create and communicate a plan of care that ensures the safe and effective collection of clinical research data (Item 2)	15	1.000
Coordinates study visits for research participants (Item 3)	14	0.933
Provides nursing leadership within the interdisciplinary team (Item 4)	14	0.933
Coordinates interdisciplinary meetings and activities within the context of the study (Item 5)	15	1.000
Coordinates referrals of patients to interdisciplinary services outside the research team	12	0.800
Communicates the impact of study procedures to research participants (Item 6)	14	0.933
Facilitates research participants' questions and concerns (Item 7)	13	0.866

Provides indirect nursing care (e.g.. participating in clinical. unit. and/or protocol rounds; planning study-related tests) in the context of research participation (Item 8)	14	0.933
Provides direct nursing care to research participants (e.g.. interacting with participants to deliver nursing care. carrying out research-related interventions. collecting specimens) (Item 9)	15	1.000
Provides education to research participants and their families about study participation. the patient’s current clinical condition. and/or the course of the disease (Item 10)	15	1.000
Monitors research participants and reports potential adverse events to a member of the research team (Item 11)	15	1.000
Records research data (e.g.. documenting vital signs. administering investigational products. participant responses) in approved source documents (e.g.. the medical record. data collection forms) (Item 12)	15	1.000
Shares clinical expertise and best practices related to clinical research through presentations. publications. and interactions with nursing colleagues (Item 13)	14	0.933
Takes part in querying and analyzing research data (Item 14)	14	0.933
Generates practice-focused questions as a result of new study procedures or interventions (Item 15)	15	1.000
Collaborates with the interdisciplinary team to develop innovations in care delivery that have the potential to improve outcomes and the accuracy of research data collection (Item 16)	15	1.000

Identifies questions suitable for clinical nursing research that arise from participation in the study team (Item 17)	15	1.000
Provides advanced mentoring and supervision for newly hired staff and for students participating as members of the research team (Item 18)	15	1.000
Performs secondary analyses of data to contribute to the development of new ideas (Item 19)	14	0.933
Serves as a resource for new researchers	14	0.933
Facilitates the initial and ongoing informed consent or assent process (Item 20)	15	1.000
Supports research participants in defining their reasons and goals for taking part in a study (Item 21)	15	1.000
Works with the interdisciplinary team to address ethical conflicts (Item 22)	15	1.000
Coordinates research activities to minimize risk to participants (Item 23)	15	1.000
Serves as a member of the ethics committee or clinical research office (e.g.. institutional review board) (Item 24)	14	0.933

Manages potential ethical and financial conflicts of interest for oneself (Item 25)	14	0.933
Participates in study development (Item 26)	14	0.933
Participates in recruiting research participants (Item 27)	15	1.000
Participates in screening potential research participants for eligibility (Item 28)	14	0.933
Coordinates and facilitates the collection of research specimens (Item 29)	15	1.000
Develops study-specific materials for research participant education (Item 30)	14	0.933
Performs quality assurance activities to ensure data integrity (Item 31)	15	1.000
Participates in preparing protocol-related reports for ethics committees and sponsors/clinical research organizations (Item 32)	14	0.933
Facilitates accurate communication between research sites (Item 33)	14	0.933

Facilitates communication within the research team (Item 34)	15	1.000
Contributes to the development of case report forms (Item 35)	14	0.933
Participates in setting up a study-specific database (Item 36)	14	0.933
Adheres to International Conference on Harmonisation guidelines for Good Clinical Practice (Item 37)	15	1.000
Collects data on research participants based on the study endpoints (Item 38)	15	1.000
Facilitates scheduling and coordination of study procedures (Item 39)	15	1.000
Provides nursing expertise to the research team during study development and implementation (Item 40)	14	0.933
Safeguards research participant data in line with regulatory requirements (Item 41)	15	1.000
Takes part in site visits and/or audits (Item 42)	15	1.000

Supports the development of study grants and budgets (Item 43)	14	0.933
Identifies the resources needed to provide direct and indirect care. with attention to efficiency. cost-effectiveness. and quality of services delivered (Item 44)	14	0.933
Oversees the human resources involved in the research process (Item 45)	15	1.000
Records data on approved study documents (e.g.. case report forms. research or study databases) (Item 46)	15	1.000
Facilitates the processing and handling (storage and transport) of research specimens (Item 47)	15	1.000
Identifies implications for clinical care during study development (e.g.. staff competencies and resources. equipment) (Item 48)	15	1.000
Identifies research trends and contributes to the preparation of related reports (Item 49)	14	0.933
